# Deafferented controllers: a fundamental failure mechanism in cortical neuroprosthetic systems

**DOI:** 10.3389/fnbeh.2015.00186

**Published:** 2015-07-17

**Authors:** Ferran Galán, Stuart N. Baker

**Affiliations:** Movement Laboratory, Institute of Neuroscience, Newcastle UniversityNewcastle upon Tyne, UK

**Keywords:** brain-machine interface (BMI), optimal feedback control, dynamical systems, motor control, neuroprosthetics

## Abstract

Brain-machine interface (BMI) research assumes that patients with disconnected neural pathways could naturally control a prosthetic device by volitionally modulating sensorimotor cortical activity usually responsible for movement coordination. However, computational approaches to motor control challenge this view. This article examines the predictions of optimal feedback control (OFC) theory on the effects that loss of motor output and sensory feedback have on the normal generation of motor commands. Example simulations of unimpaired, totally disconnected, and deafferented controllers illustrate that by neglecting the dynamic interplay between motor commands, state estimation, feedback and behavior, current BMI systems face translational challenges rooted in a debatable assumption and experimental models of limited validity.

## Introduction

Current BMI research (Bensmaia and Miller, 2014) aims at extracting movement parameters believed to be encoded in motor cortical areas that would enable neuroprosthetic control after peripheral disconnection. However, despite encouraging early laboratory monkey experiments (Taylor et al., 2002; Carmena et al., 2003; Moritz et al., 2008; Velliste et al., 2008; Ethier et al., 2012), cortical neuroprosthetic control (Hochberg et al., 2012; Collinger et al., 2013) is poor compared to natural movements and highly dependent on visual feedback. Understanding the mechanisms underlying poor BMI performance is the key to clinical translation; currently funded efforts focus on identifying failure mechanisms associated with degraded neural signal acquisition from chronically implanted electrode arrays, unreliable motor decoding of such neural signals, and delivery of poor sensory feedback (Miranda et al., 2015). This article considers a fundamental failure mechanism largely underappreciated by the BMI community: the assumption that sensorimotor cortical activity engaged during unimpaired movement remains available for prosthetic control after peripheral disconnection.

Especially relevant to BMI research, the OFC theory of motor coordination (Todorov and Jordan, 2002) highlights the dynamic interplay between motor commands and sensory feedback, discouraging ideas of neural coding/decoding and emphasizing neural dynamics when interpreting sensorimotor function (Scott, 2012; for a recent review of OFC and its possible biological underpinnings see Scott et al., 2015; Shenoy et al., 2013). This is supported by work demonstrating the influence of sensory feedback on ongoing M1 activity (Herter et al., 2009; Suminski et al., 2010; Pruszynski et al., 2011), studies describing changes in neural tuning at different time scales (Sergio et al., 2005; Hatsopoulos et al., 2007; Churchland et al., 2012) and under different feedback conditions (Gaunt et al., 2013), and work showing altered EEG movement-related information in the absence of kinaesthetic feedback (Galán et al., 2015).

The OFC framework further predicts that loss of motor output and sensory feedback will have an impact on the normal generation of motor commands; although these might still be initiated, they are likely to be altered compared with those responsible for producing unimpaired movement. Such a prediction poses relevant questions to BMI research that need addressing: is it possible at all after peripheral disconnection to use sensorimotor cortical activity for reliable cortical neuroprosthetic control? If not, what is needed? And, in such a scenario, how can studies with able-bodied experimental subjects contribute to advancing the development of reliable BMI systems? The following sections illustrate the predictions of an infinite-horizon OFC model (Qian et al., 2013) in situations with different types of peripheral disconnection, and discuss the implications for the BMI field.

## Peripheral disconnections in OFC

**Figure 1:**
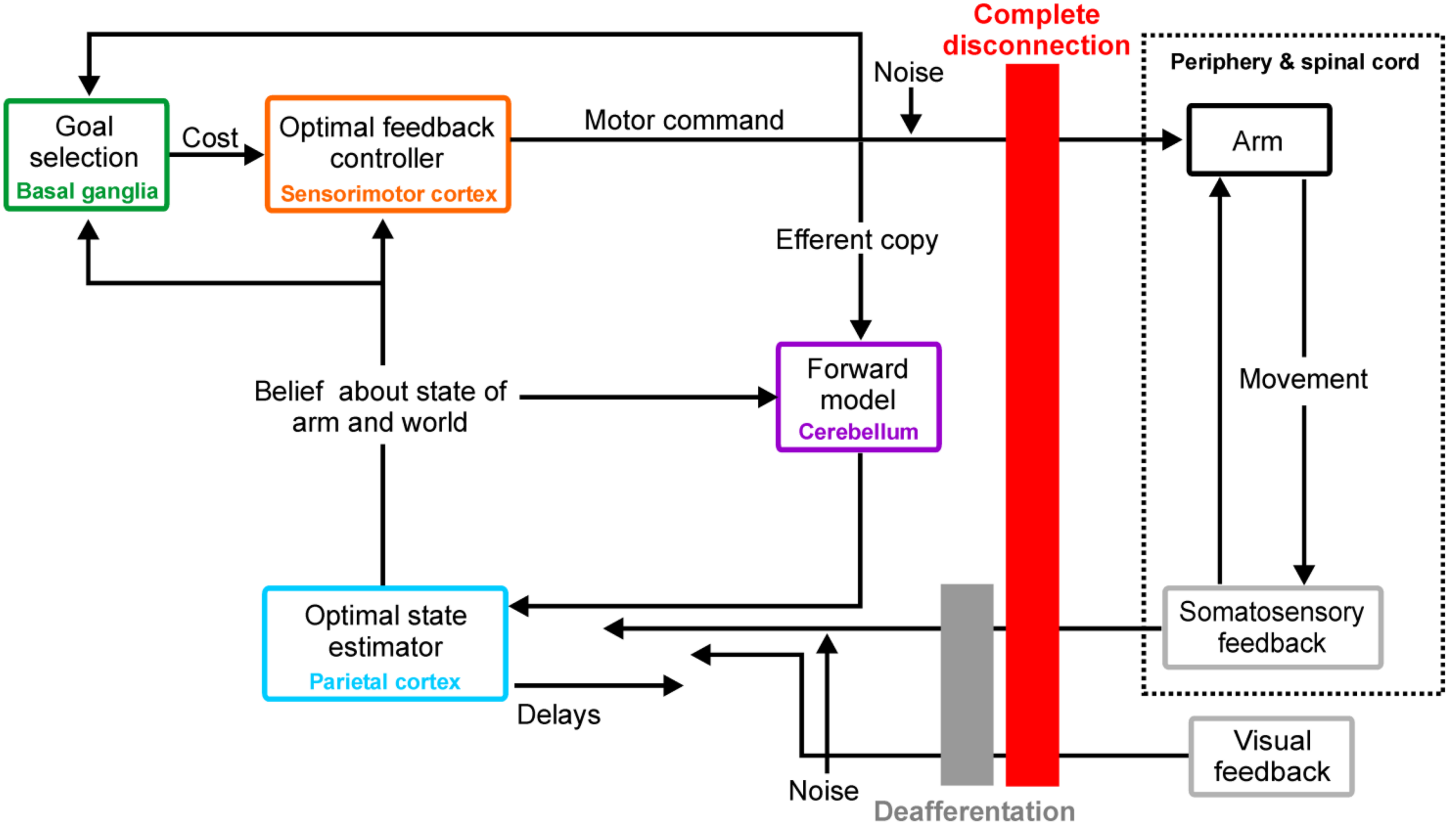
**Optimal feedback control, putative neural substrates, and peripheral disconnections**. Diagram illustrating OFC framework, its possible neural basis and both simulated peripheral disconnections: complete disconnection (red) and deafferentation (gray). See section “Peripheral disconnections in OFC” for details.

Figure 1 illustrates the OFC framework. To achieve a goal such as an arm movement, optimal feedback controllers generate motor commands that minimize a cost function (which might include error and energy expenditure) based on their belief about the current state of the arm and the world. Such a belief is formed by integrating through an optimal estimator the state changes observed through delayed afferent feedback with those predicted by forward models, which use knowledge about the system dynamics acting on a copy of the motor commands. Note that motor commands and sensory feedback are both susceptible to corruption by noise. References (Shadmehr and Krakauer, 2008; Scott, 2012; Shadmehr and Mussa-Ivaldi, 2012; Scott et al., 2015) provide further detail on the computational and neural basis of the OFC framework in voluntary motor control.

Understanding the causal links between different movement phases such as preparation and execution is especially relevant to cortical neuroprosthetic control (Shenoy et al., 2011). An interesting property of the infinite-horizon OFC model utilized here (Qian et al., 2013) is the integration of movement control and postural maintenance, without artificial separations. This occurs through the application of steady-state solutions for state estimation, movement execution and postural maintenance. The state estimator and controller are computed in a preparatory phase before movement starts, but motor commands can only be determined during the movement attempt as they depend on estimated states and sensory feedback. Therefore, movement properties such as duration and trajectory, as well as the motor commands themselves, emerge from the model with inherent trial-to-trial variability. In brief, Qian et al. (2013) consider a linear dynamical system governed by stochastic differential equations:

(1) dx=(Ax+φBu)dt+Fxdβ+Yudγ+Gdω,

(2) dy=Cxdt+Ddξ,

where *x* is the state *n*-vector, *u* is the control *k*-vector (motor command), and *y* is the sensory feedback vector. β and γ are scalar Wiener processes, and ω and ξ are *n*- and *k*-vector Wiener processes modeling noise in control and sensory feedback, all independent from each other and standardized so that over a time step *dt*, the corresponding Gaussian white noise processes have variance *dt*. *A, B, C, D, F, G* and *Y* are constant matrices; *A* and *B* define the motor plant according to Newtonian mechanics and the muscle force equation, *C* and *D* define observation and observation noise, *F* and *Y* define state- and control-dependent noise (multiplicative or signal dependent noise), and *G* defines control-independent noise. φ is a scalar modeling efferent disconnection: if the system remains intact then φ = 1, if otherwise motor commands are blocked then φ = 0. The current state *x* is not available for control and is estimated according to a linear Kalman filter (Kalman, 1960):

(3) $$d\hat{x}=\left(A\hat{x}+Bu\right)dt+K\left(dy-C\hat{x}dt\right),$$

where the first term is the prediction based on a forward internal model and an efference copy of the control signal, and the second term is the correction based on the discrepancy between observed and predicted sensory feedback.

The motor command is a linear function of $\hat{x}$:

(4) $$u=-L\hat{x},$$

and estimator gain *K* and controller *L* are determined by optimizing costs. According to Phillis's infinite-horizon formulation (Phillis, 1985), the estimator cost is defined as the steady-state variance of the estimation error $\tilde{x}\equiv x-\hat{x}$,

(5) $$J_{1}=\underset{t\to \infty }{\lim}E\left[{\tilde{x}}^{T}U\tilde{x}\right],$$

and the controller cost as the steady-state cost per unit of time,

(6) $$J_{2}=E\left[\underset{t\to \infty }{\lim}\frac{1}{t}\int_{0}^{t}\left(x^{T}Qx\text{+}u^{T}Ru\right)dt\right],$$

where the first term measures accuracy of the reaching target, the second term measures energetic cost, and matrices *U*, *Q*, and *R* are constant and symmetric.

The simulations reproduced *unimpaired* single-joint reaching movements of the forearm at the elbow as in (Qian et al., 2013), but additionally tested two different types of peripheral disconnection (see Figure 1): complete disconnection (*disconnected*, *K* = φ = 0) and deafferentation (*deafferented*, *K* = 0). First, estimator gain *K* and controller *L* were numerically obtained from an *unimpaired* controller as in (Qian et al., 2013); note that they only depend on plant and cost parameters. Then, optimal steady state estimator gain *K* and controller *L* were applied to move the hand (*n* = 20 trials) toward a target placed 50 cm away according to the system dynamics defined by *unimpaired, disconnected*, and *deafferented* conditions. (Matlab code obtained from N. Qian upon request and modified to simulate deafferentation and complete disconnection).

**Figure 2:**
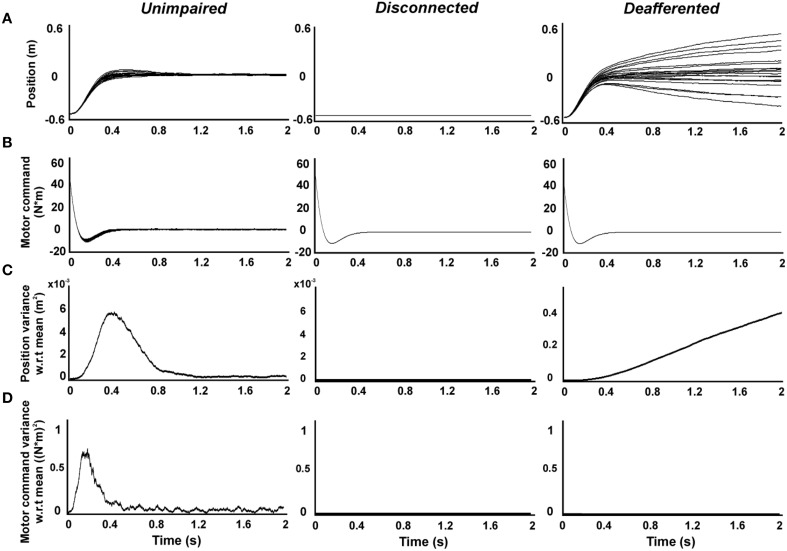
**Simulations of reaching movements produced by** ***unimpaired***, ***disconnected***, **and** ***deafferented*** **controllers**. Simulated single-joint reaching movements of the forearm (*n* = 20) toward a target placed 50 cm from starting position. **(A)** Position **(B)** motor command **(C)** position variance with respect to the average. **(D)** Motor command variance with respect to the average.

Figure 2 shows time-resolved profiles of hand position, motor command, and their variance with respect to the average, generated by the system dynamics defined by *unimpaired, disconnected, and deafferented* controllers. The model reproduced typically-observed properties of *unimpaired* movements with trial-to-trial variability (Qian et al., 2013) (Figures 2A–D, *unimpaired*). By contrast, the *disconnected* controller generated (Equation 4) highly stereotyped (Figures 2B,D; *disconnected*) motor commands based on estimates that solely rely on the predictions of a forward model using efference copy (Wolpert and Miall, 1996; Wolpert et al., 1998) (note that *K* = 0 in Equation 3). Such motor commands generated no overt output, however, due to the efferent block (Figures 2A,C; *disconnected*).

These results seem to suggest that not only is the motor command intact in the *disconnected* state, but it is actually less variable than in the *unimpaired* condition. Does this mean that decoding of the motor command would lead to effective movements of a neural prosthetic? Such decoding can be modeled using a *deafferented* controller, where efferents remain connected, revealing what movements would be generated from the stereotyped command seen in the *disconnected* state. This revealed highly variable movement trajectories, resulting from the accumulation of errors over time in the state estimate which must rely, in the absence of sensory feedback, on only forward model predictions from efference copy. The monotonic increase of state variance reproduced movement profiles displayed by patients suffering from sensory neuropathies without visual feedback (Sanes et al., 1984; Gordon et al., 1995) (Figures 2A,C; *deafferented*). Paradoxically, the generation of motor commands with trial-to-trial variability ensures successful movements (Todorov and Jordan, 2002) (Figure 2 *unimpaired*), whereas the lack of such variability ensures movement imprecision (Figure 2 *deafferented*). These examples illustrate that the lack of sensory feedback from the periphery suffered by *disconnected* and *deafferented* controllers has an impact on the generation of motor commands via poor state estimation. Execution of such motor commands (e.g., by a decoder driving a neural prosthetic) unavoidably leads to poor motor control.

## Discussion

Current BMI systems aim to extract neural information from sensorimotor cortical areas to enable prosthetic control to the paralyzed. However, the above examples illustrate that these areas, considered as optimal feedback controllers (see Figure 1; Shadmehr and Krakauer, 2008; Scott, 2012), are likely to generate motor commands leading to poor prosthetic control when disconnected from the periphery. An ideal bidirectional biomimetic approach which restored both sensory and motor function would theoretically allow for feedback-corrected state estimation and restore reliable control; recent work on hand prosthetics enabling effective modulation of grasping force by conveying nearly “natural” sensory feedback (Raspopovic et al., 2014) supports this notion. However, such interfacing at higher levels of the neuraxis is beyond current technical developments (Bensmaia and Miller, 2014). More feasible might be an implementation of bidirectional interfacing based on learned non-biomimetic associations (Bensmaia and Miller, 2014). Studies with able-bodied experimental subjects demonstrating volitional control of sensorimotor cortical activity (Fetz, 1969), neural adaptation during efferent BMI control (Jarosiewicz et al., 2008; Ganguly and Carmena, 2009) and discrimination of evoked percepts by intracortical microstimulation (ICMS) to somatosensory cortex (Romo et al., 1998; London et al., 2008; O'Doherty et al., 2011) all support that this approach may have merit, although it remains unknown whether this will deliver sufficiently rich sensory feedback to enable precise cortical neuroprosthetic control. At present, deafferented patients who only rely on visual feedback appear to provide an upper bound on current cortical neuroprosthetic systems' performance.

In Figure 1 we have assigned putative neural structures to each component of the OFC model. In reality the system is likely to be distributed, with a single box in that diagram implemented by multiple neural circuits at different levels of the neuraxis. Feedback is also distributed, being received for example by both spinal and cortical circuits. One consequence of such an arrangement may be that the full motor command can never be read out from one neural structure on its own.

How do the ideas presented above fit with experimental results from the BMI field? Two FDA clinical trials involving patients with paralysis (Hochberg et al., 2012; Collinger et al., 2013) showed impoverished cortical control compared to natural movements, in agreement with these concepts. Other than these reports, the majority of BMI work involve able-bodied non-human primates (Shenoy and Carmena, 2014), with intact sensory feedback loops from the periphery. In just a few instances was peripheral disconnection modeled with nerve blocks at upper arm (Moritz et al., 2008) and elbow (Pohlmeyer et al., 2009; Ethier et al., 2012), but even in those cases feedback above the block was intact. The ability to use residual sensorimotor loops has been previously demonstrated in amputees (Kuiken et al., 2007; Raspopovic et al., 2014), where access to motor representations of the missing limb appears conditional upon the re-establishment of peripheral connections (Reilly et al., 2006). Evidence for effective neuroprosthetic systems based on extracting control signals from sensorimotor cortical areas totally disconnected from the periphery is currently lacking. We argue that neuroprostheses which seek to exploit preserved sensorimotor loops may offer the most promising control alternatives on theoretical grounds.

### Conflict of interest statement

The authors declare that the research was conducted in the absence of any commercial or financial relationships that could be construed as a potential conflict of interest.
